# Histopathology and biochemistry analysis of the interaction between sunitinib and paracetamol in mice

**DOI:** 10.1186/1471-2210-10-14

**Published:** 2010-10-15

**Authors:** Adeline YL Lim, Ignacio Segarra, Srikumar Chakravarthi, Sufyan Akram, John P Judson

**Affiliations:** 1 Department of Human Biology, School of Medicine, International Medical University, Jalan 19/155B, Bukit Jalil, 57000 Kuala Lumpur, Malaysia; 2 Department of Pharmaceutical Technology, School of Pharmacy and Health Sciences, International Medical University, Jalan 19/155B, Bukit Jalil, 57000 Kuala Lumpur, Malaysia; 3 Department of Pathology, International Medical University, Jalan 19/155B, Bukit Jalil, 57000 Kuala Lumpur, Malaysia

## Abstract

**Background:**

Sunitinib, a tyrosine kinase inhibitor to treat GIST and mRCC may interact with paracetamol as both undergo P450 mediated biotransformation and P-glycoprotein transport. This study evaluates the effects of sunitinib-paracetamol coadministration on liver and renal function biomarkers and liver, kidney, brain, heart and spleen histopathology. ICR male mice (n = 6 per group/dose) were administered saline (group-A) or paracetamol 500 mg/kg IP (group-B), or sunitinib at 25, 50, 80, 100, 140 mg/kg PO (group-C) or coadministered sunitinib at 25, 50, 80, 100, 140 mg/kg PO and paracetamol IP at fixed dose 500 mg/kg (group-D). Paracetamol was administered 15 min before sunitinib. Mice were sacrificed 4 h post sunitinib administration.

**Results:**

Group-A serum ALT and AST levels were 14.29 ± 2.31 U/L and 160.37 ± 24.74 U/L respectively and increased to 249.6 ± 222.7 U/L and 377.1 ± 173.6 U/L respectively in group-B; group-C ALT and AST ranged 36.75-75.02 U/L and 204.4-290.3 U/L respectively. After paracetamol coadministration with low sunitinib doses (group-D), ALT and AST concentrations ranged 182.79-221.03 U/L and 259.7-264.4 U/L respectively, lower than group-B. Paracetamol coadministration with high sunitinib doses showed higher ALT and AST values (range 269.6-349.2 U/L and 430.2-540.3 U/L respectively), p < 0.05. Hepatic histopathology showed vascular congestion in group-B; mild congestion in group-C (but lesser than in group-B and D). In group-D, at low doses of sunitinib, lesser damage than in group-B occurred but larger changes including congestion were observed at high sunitinib doses. BUN levels were higher (p < 0.05) for group-B (33.81 ± 5.68 mg/dL) and group-D (range 35.01 ± 6.95 U/L to 52.85 ± 12.53 U/L) compared to group-A (15.60 ± 2.17 mg/dL) and group-C (range 17.50 ± 1.25 U/L to 26.68 ± 6.05 U/L). Creatinine remained unchanged. Renal congestion and necrosis was lower in group-C than group-B but was higher in group-D (p > 0.05). Mild cardiotoxicity occurred in groups B, C and D. Brain vascular congestion occurred at high doses of sunitinib administered alone or with paracetamol. Hepatic and renal biomarkers correlated with histopathology signs.

**Conclusions:**

Paracetamol and sunitinib coadministration may lead to dose dependent outcomes exhibiting mild hepatoprotective effect or increased hepatotoxicity. Sunitinib at high doses show renal, cardiac and brain toxicity. Liver and renal function monitoring is recommended.

## Background

Drug-drug interactions (DDIs) defined as an increase or decrease in the clinical effect of a given drug due to interference by another drug, is a significant cause of morbidity and mortality worldwide [1]. DDIs may result in adverse clinical events, by decreasing the therapeutic effect of a drug or by enhancing drug toxicity [2].

Cancer patients present high risk of DDIs as polypharmacy for the treatment of cancer as well as other related syndromes is common [3]. They are also very susceptible to pain, with moderate or severe pain prevalent in at least 50% of cancer patients [4,5]. Severe DDIs have been observed between anti-cancer and pain management drugs. Some patients treated with imatinib, a generally well-tolerated chemotherapeutic agent, have experienced renal and hepatic toxicity, which was increased and fatal in some cases upon coadministration with paracetamol [6]. Mechanistic studies in animal models showed changes in imatinib pharmacokinetic and tissue penetration profiles [7] but most importantly, an increased of irreversible hepatotoxicity was observed when both drugs were co-administered [8]. The importance of interactions with paracetamol is also relevant to sunitinib: a patient with relapsed metastatic gastrointestinal stromal tumour (GIST) treated with sunitinib and taking also paracetamol and levothyroxine, developed acute liver failure with fatal outcome [9].

Sunitinib (sunitinib malate; SU11248, SUTENT^®^) is a novel oral multitargeted tyrosine kinase inhibitor that received regular approval from the United States FDA for the treatment of GIST as well as advanced renal cell carcinoma (RCC) after progression [10] or intolerance to imatinib mesylate [11,12]. Sunitinib inhibits various receptor tyrosine kinases such as the vascular endothelial growth factor receptors (VEGFR) [13], the foetal liver tyrosine kinase receptor 3 (FLT3) [14], stem-cell factor receptor (c-KIT) [15], platelet-derived growth factor receptors PDGFRα and PDGFRβ [16], and colony stimulating factor type 1 receptor (CSF-1R) [17]. Consequently, there is inhibition of angiogenesis, tumor growth and metastasis [18,19].

In humans, the maximum plasma concentration is reached 6-12 h after dosing, shows good tissue distribution, dose proportionality at the range 25-100 mg, and is highly bound to albumin (95%). Sunitinib is metabolized primarily by the cytochrome P450 3A4 to form main active metabolite SU12662 that is further metabolized by CYP3A4 [11]. Sunitinib and its metabolite, which is also highly bound to plasma proteins (90%), have half-lives of 40-60 h and 80-110 h respectively, 61% of the sunitinib dose is eliminated in the faeces and around 16% is recovered unchanged in urine [11,17]. Pharmacokinetic studies in mice have shown that sunitinib is readily absorbed, presents dose proportionality and the maximum concentration is achieved within 0.5 to 6 h. Both sunitinib and the main metabolite (SU12662) are highly bound to mouse plasma proteins (91% and 95% respectively) with the fraction unbound independent of the concentration. The elimination half-life in mice is 1.5 to 7.6 h after oral administration [20].

Although the therapeutic benefits of sunitinib are great, sunitinib treatment has a significant number of side effects including fatigue [21], hypertension [11,22], cardiac dysfunction [23], thyroid dysfunction [23], hand food skin reaction [19,24], hair depigmentation [19], asthenia [25], haematologic toxicity [11], and less commonly posterior reversible encephalopathy [26], cardiotoxicity and hypothyroidism [27] and tumour lyses syndrome [28]. In addition, sunitinib treatment also caused treatment-emergent laboratory abnormalities such as a rise in serum alanine transaminase (ALT), aspartate transaminase (AST) as well as serum creatinine concentrations in GIST and mRCC patients which was mainly Grade 1 and 2 in severity [11,12].

Sunitinib is likely to present a variety of metabolism based drug-drug interactions Biotransformation of sunitinib to form the pharmacologically active N-desethyl metabolite, SU12662 is affected by inhibitor or inducers of CYP3A4 [11]. Strong CYP3A4 inhibitors such as ketoconazole have shown to decrease the metabolism of sunitinib and to increase plasma sunitinib concentrations [13]. Similarly, CYP3A4 inducers (e.g. rifampin) may decrease sunitinib plasma concentrations when the inducer and sunitinib are co-administered together which lead to subtherapeutic sunitinib levels [20]. Furthermore, there are some indications that CYP1A1 and 1A2 may also play a role in sunitinib biotransformation [29]. Thus, if it is necessary to concurrently administer sunitinib and a CYP3A4 inhibitor or inducer, it is recommended that the dose of sunitinib should be adjusted (decreased or increased) and that diligent monitoring for toxicity be carried out [11].

Given the nature of the cancer treatment, cancer patients are likely to be administered pain management drugs [2,4]. In this study, we used a mouse model to evaluate the safety and toxicity upon the drug-drug interaction between sunitinib and paracetamol, a widely used over the counter analgesic and antipyretic drug. Though, paracetamol is regarded as a safe drug at therapeutic doses; at larger or chronic doses may result in severe liver and renal injuries [30], including changes in alkaline phosphatase (ALP), AST, ALT and creatinine plasma levels [31] which could be additive to those of sunitinib [10]. Small amounts of paracetamol (~5%) undergo P450 mediated oxidation to a reactive electrophilic intermediate, N-acetyl-p-benzoquinoneimine (NAPQI) [32]. Several P450 isoforms including CYP3A1, 2E1, 1A2 and 2D6 are implicated in the activation of paracetamol to NAPQI in both humans and rodents [33]. Furthermore to the hepatotoxicity, potential overlapping sunitinib-paracetamol toxicity could also include renal insufficiency [29] and cardiotoxicity [34].

The current study aims to evaluate the toxicity associated with the coadministration of sunitinib and paracetamol in mice. A histopathology assessment of the liver, kidney, spleen, heart and brain is carried out and correlated with the plasma levels of the biochemical markers AST, ALT, creatinine and urea.

## Methods

### Materials

Stock solution of paracetamol (Fluka, France) was prepared in reversed osmosis (RO) water at 30 mg/mL. The paracetamol solution was vortex mixed, sonicated (25 min) and kept warm briefly (50°C) prior to IP administration to mice. Sunitinib malate (Zhejiang Esun Chemical Co. Ltd., China) was diluted in RO water to 15 mg/mL, vortex mixed, sonicated (25 min), protected from light at room temperature until PO administration.

### Animals and experimental protocols

Male ICR mice of similar age (8-12 weeks) and weight (25-35 g) were obtained from University Putra Malaysia and housed at the International Medical University (IMU) animal holding facility with 12 h light cycles at 20 ± 2°C for acclimatization. The experimental animals were provided food and water *ad libitum*. All animal procedures had been reviewed and approved by the IMU Institutional Animal Use and Ethics Committee preceding the initiation of this study.

Animals were fasted overnight prior to dose administration and randomly assigned to each of the four experimental groups. Mice in group A (n = 6) were administered saline; mice in group B mice (n = 6) were administered paracetamol only, 500 mg/kg IP. Group C was further subdivided into five different dose-groups of sunitinib only treatment: 25, 50, 80, 100 and 140 mg/kg administered PO (n = 6 for each dose group). Finally, mice in group D were co-administered paracetamol (500 mg/kg IP) and sunitinib at different doses: 25, 50, 80, 100 and 140 mg/kg PO (n = 6 each dose group). A feeding needle was used to ensure the full administration of the dose. Paracetamol was administered 15 minutes before sunitinib and animals were sacrificed 4 hours post sunitinib administration by cervical dislocation. Blood was obtained via cardiac puncture, allowed to clot, centrifuged (1500 rpm, 10 min, room temperature) and stored at -20°C until analysis. The liver and kidneys were excised from the mice and processed for histology assessment.

### Serum biomarkers analysis

Biochemistry analysis of serum ALT and AST was performed using standard assay kits (BiooScientific Corp., USA). The concentration in duplicate samples was measured at 340 nm using a microplate reader (Tecan Infinite F200). Similarly, blood urea nitrogen (BUN) and creatinine concentration were measured using specific assay kits (BioAssay Systems, USA) and measured at 490 nm (duplicate samples) in a Dynex OpsysMR microplate reader.

The effect of the drug combination on the biomarker levels compared to sunitinib alone treatment was assessed with the aid of SPSS 16.0 with the significance level set at p < 0.05. Independent-samples t-test (for samples normally distributed based on Shapiro-Wilk test) for pair-wise comparison was performed at each dose of the sunitinib alone and sunitinib and paracetamol combination groups. Samples that were not normally distributed were tested using the Mann Whitney test.

### Histopathology assessment of tissues

Processing of tissue samples for histology assessment followed established procedures. In brief, the tissue samples were rinsed with 0.9% saline solution, fixed in 10% formalin. Then the diagonal section of the liver, the transverse section of the kidneys and heart and the horizontal section of the spleen and the posterior section of the brain were obtained and processed (Leica TP1020, Japan) as follows: (1) 10% neutral buffered formalin for 1 h, twice; (2) 70% alcohol for 1.5 h; (3) 80% alcohol for 1.5 h; (4) 90% alcohol for 1.5 h; (5) absolute alcohol for 1.5 h, twice; (6) xylene for 1.5 h, twice; (7) in molten wax at 65°C for 2.5 h two changes. The processed tissues were embedded in paraffin and sectioned at 4 microns thickness, placed on frosted glass slides and dried on a 70°C hot plate for 30 minutes.

The tissues were stained using the hematoxylin and eosin (H&E) stains. The sections were dewaxed in two changes of xylene (3 min each), hydrated in two changes of 100% ethanol, followed by 90% ethanol and 70% ethanol, for 3 min each, rinsed with water (3 min) and stained. The stained tissues were dehydrated with 70% ethanol followed by 90% ethanol, placed in two changes of 100% ethanol for 3 minutes each and cleaned with two changes of xylene (3 min each).

Histopathology changes were observed and grouped based on two main criteria: vascular changes including vessel congestion, extravasation of red blood cells and hematoma formation; and necrotic changes including necrosis, fibrosis, nuclear changes, abscesses and cell regeneration. The morphological changes were assessed semi-quantitatively, blind by two independent assessors and graded as follows: No change - 0 (no distinguishable change, 0%); mild change - 1 (initiation of changes, up to 30%); moderate change - 2 (patent changes, 31-60%); severe change - 3 (wide spread changes, 61-100%) [35]. Then, using SPSS 16.0, the Mann-Whitney test was used for pair-wise comparison between the sunitinib alone group and the combination group at each dose. The differences were considered significance if p < 0.05.

## Results

The changes in toxicity associated with the coadministration of sunitinib and paracetamol were assessed at biochemistry and histopathology level. The serum concentrations of the biochemical markers ALT, AST, urea and creatinine were obtained to evaluate the liver and renal functions. In addition, the histopathological changes in the target organs such as liver, kidneys, heart, brain and spleen were semi-quantitatively graded.

### Liver and renal function biomarkers

**Figure 1:**
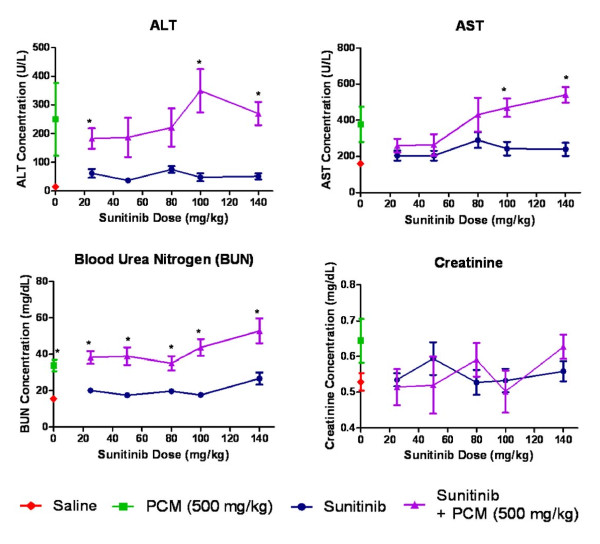
**Liver and renal function biomarkers**. Change in biomarkers upon administration of saline vehicle (baseline control), paracetamol, sunitinib at several doses or combination treatment paracetamol and sunitinib. (* p < 0.05 bewteen groups C and D based on t-test except for ALT that Mann Whitney test was used).

The mean serum AST level in untreated mice (Table 1), vehicle control group was 160.37 ± 24.74 U/L but it was 2-fold greater (377.09 ± 173.55 U/L) in mice treated with paracetamol (Figure 1). Mice treated with increasing doses of sunitinib showed a gradual elevation of AST serum concentration (range 204.64 - 290.28 U/L) following rising sunitinib doses. However, the pattern observed upon coadministration of sunitinib and paracetamol was different. At lower doses of sunitinib, the mean AST serum concentrations were slightly higher than those of the sunitinib alone group, but lower than the concentration found upon administration of paracetamol alone: 31.1% and 29.9% for the 25 mg/kg and 50 mg/kg dose respectively. However, a large increase in AST concentrations were observed at the higher doses of sunitinib coadministered with paracetamol which was statistically significant at 100 mg/kg (p < 0.01) and 140 mg/kg (p < 0.001) doses.

**Table 1:** Mean ± SE serum biomarker concentration for each of the study arms.

Group	Dose (mg/kg)		AST	ALT	BUN	Creatinine
					
	SUN	PCM	(U/L)	(U/L)	(mg/dL)	(mg/dL)
Vehicle	-	-	160.4 ± 24.7	14.29 ± 2.31	15.6 ± 2.17	0.529 ± 0.044

PCM	-	500	377.1 ± 173.6	249.6 ± 222.7	33.81 ± 5.68	0.644 ± 0.110

Sunitinib	25	-	204.6 ± 50.3	61.40 ± 26.57	20.08 ± 2.54	0.535 ± 0.033
	50	-	204.4 ± 49.4	36.75 ± 12.15	17.5 ± 1.25	0.594 ± 0.082
	80	-	290.3 ± 77.7	75.02 ± 20.37	19.71 ± 0.58	0.528 ± 0.064
	100	-	243.0 ± 66.4	47.61 ± 24.50	17.62 ± 0.94	0.533 ± 0.060
	140	-	239.5 ± 65.9	50.89 ± 18.90	26.68 ± 6.05	0.559 ± 0.051

Sunitinib	25	500	259.7 ± 67.4^+^	182.8 ± 64.91*	38.24 ± 6.08*	0.514 ± 0.089
& PCM	50	500	264.4 ± 104.9	186.7 ± 124.3	38.89 ± 8.69*	0.520 ± 0.143
	80	500	430.2 ± 163.6	221.0 ± 113.0	35.01 ± 6.95*	0.591 ± 0.084
	100	500	469.8 ± 90.3^+^*	349.2 ± 132.4*	43.75 ± 8.37*	0.502 ± 0.103
	140	500	540.3 ± 76.1^+^*	269.6 ± 72.6*	52.85 ± 12.53^+^*	0.627 ± 0.060

+ p < 0.05 in comparison with group B (paracetamol)

* p < 0.05 in comparison with group C (sunitinib alone)

Similarly, the serum ALT concentrations were measured (Figure 1, Table 1). In the vehicle control group the ALT serum concentration was 14.29 ± 2.31 U/L and it slightly increased in the sunitinib treatment group (range 36.75 - 75.02 U/L). As expected, a large increase in serum ALT levels was observed after administration of paracetamol only (249.60 ± 222.72 U/L) although it did not reach statistical difference with the baseline control value, probably due to the large variability. Parallel to AST, there was a significant ALT elevation in the coadministration group at 100 mg/kg (p < 0.05) and 140 mg/kg (p < 0.01) doses of sunitinib. In addition, at lower doses of sunitinib with paracetamol coadministration, the ALT levels were found lower than those in the group that received paracetamol only: at 25 mg/kg the ALT concentration was 26.8% lower and at 50 mg/kg it was 25.2% lower. However, it did not reach statistical significance probably due to the variability in the paracetamol group.

Renal biomarkers included the blood urea nitrogen (BUN) and creatinine (Figure 1, Table 1). The BUN concentrations were slightly increased (range 17.50 - 26.68 mg/dL) after administration of sunitinib in comparison to those of baseline control group (15.60 ± 2.17 mg/dL) with significant differences (p < 0.05) with the control group at 25, 80 and 140 mg/kg sunitinib doses. A significant increase in BUN concentration was observed in the group administered paracetamol only (33.81 ± 5.68 mg/dL, p < 0.05). In addition, all dose groups of the coadministration group showed differences with the respective sunitinib group dose (p < 0.05). As for creatinine concentrations (Figure 1), there was no significant difference between the serum concentrations in the baseline control group (0.529 ± 0.044 mg/dL) and those found in mice treated with increasing doses of sunitinib alone (range 0.528 - 0.594 mg/dL). However, administration of paracetamol alone increased 21% the creatinine serum concentration. Finally, the combination treatment sunitinib-paracetamol did not show any significant rise in mean creatinine concentrations.

### Histopathology in liver tissue

**Figure 2:**
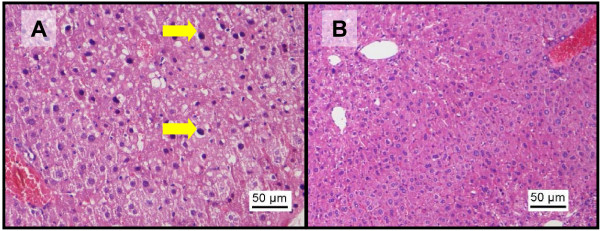
**Histopathological changes in liver after administration of paracetamol**. Representative microphotographs of liver tissue sections (H&E staining, 200x) showing (A) nuclear pyknosis (arrows), vascular congestion and fatty change in liver parenchyma and (B) areas of centrilobular necrosis and vascular congestion involving the portal triad and dilation of central vein indicating backflow of circulation.

**Figure 3:**
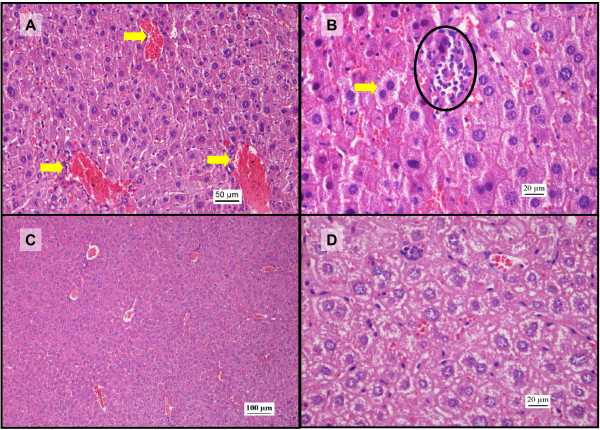
**Histopathological changes in liver after administration of sunitinib (H&E staining)**. A: Photomicrograph (200x) of liver section after administration of 25 mg/kg sunitinib showing vascular congestion (arrows) in the central veins and red blood cells pooling in the sinusoids. B: Liver section (400x) after administration of sunitinib 25 mg/kg. Notice a microabscess (circle) involving a few hepatocytes with inflammatory cells and necrotic debris. Hepatocytes undergoing cytoplasmic condensation and regeneration are also observed (arrow). C: Photomicrograph (100x) from the sunitinib 80 mg/kg group showing vascular congestion involving the central veins amidst hepatocytes surrounded by dilated sinusoids. D: Photomicrograph (400x) from the sunitinib 100 mg/kg group. Notice the hepatocytes in various stages of cytoplasmic condensation, suggesting early stages of cell injury.

**Figure 4:**
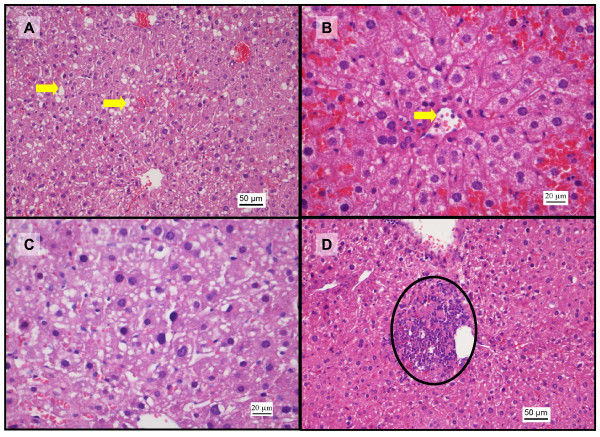
**Histopathological changes in liver after coadministration of sunitinib and paracetamol (H&E staining)**. Photomicrographs of liver sections after coadministration of sunitinib (25 mg/kg) and paracetamol. A: (200x) damaged hepatocytes with areas of fatty change (arrows), sinusoid congestion and central vein dilation. B: (400x) red blood cells accumulated around the draining pathways of the central vein (arrow) suggesting backflow congestion. C: fatty change in the cytoplasm and pyknotic nuclei (arrows). D: photomicrograph (200x) after combination treatment of sunitinib 50 mg/kg and paracetamol showing a focal area of microabscess (circle) consisting of inflammatory cells and necrotic debris.

**Figure 5:**
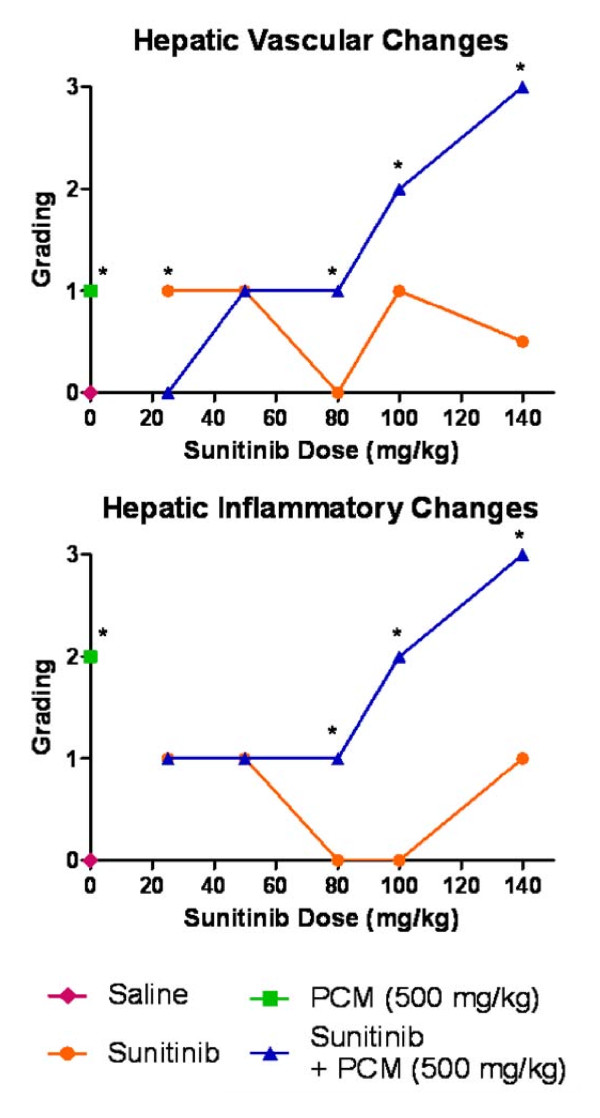
**Hepatic vascular and inflammatory changes**. Graphical representation of the score and grading of the vascular and inflammatory changes observed in the liver after administration of saline vehicle (baseline control), paracetamol, sunitinib at several doses or combination treatment paracetamol and sunitinib (* p < 0.05 bewteen groups C and D based on Mann Whitney test).

The histopathology assessment in liver was performed for all groups. Mice in the vehicle (saline) control group showed normal, well defined histological structures without any signs of vascular or inflammatory changes. The histopathology analysis of the liver revealed signs of toxicity after administration of paracetamol. This toxicity was significant (p < 0.05) in comparison with baseline group and included mild vascular congestion and moderate inflammatory changes with congested sinusoids, nuclear changes, and centrilobular necrosis (Figure 2). Treatment with increasing doses of sunitinib alone resulted in mild vascular and inflammatory changes (not significant in comparison with baseline control) including microabscess formation and cytoplasmic condensation indicating early cellular injury (Figure 3). In the combination treatment, there was moderate to severe vascular and sinusoidal congestion, as well as inflammatory changes with extensive necrosis at high doses of sunitinib that were statistically significant in comparison with the sunitinib alone group (p < 0.05) at 80, 100 and 140 mg/kg (Figure 4 and 5). However, at lower doses of sunitinib coadministered with paracetamol, there were mild vascular changes comparable to those in the group treated with paracetamol and mild inflammatory changes which were even less severe than those observed after paracetamol administration (Figure 5).

### Renal histopathology

**Figure 6:**
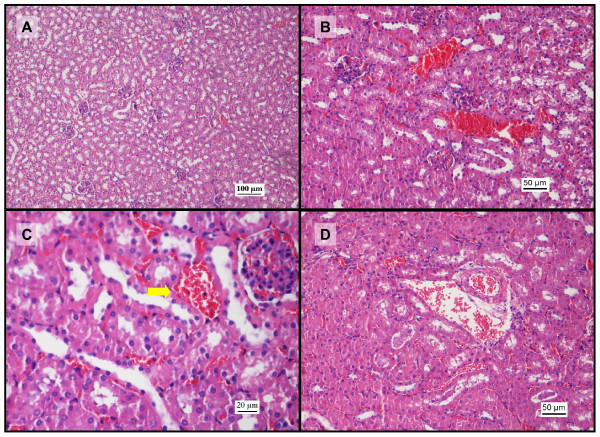
**Histopathological changes in kidney after administration of paracetamol (H&E staining)**. Photomicrographs of kidney tissue sections after administration of vehicle or paracetamol. A: (100x) saline group showing normal orientation of nephrons with adequate glomeruli and well spaced tubules. B: Photomicrograph (200x) showing areas of red blood cells extravasating into the interstitium and amidst the spaces between the tubules. C: photomicrograph (400x) showing congested vasculature (arrow) in the juxtaglomerular spaces. D: photomicrograph (200x) where dilated spaces filled with accumulated blood cells, suggesting focal haemorrhages can be observed.

**Figure 7:**
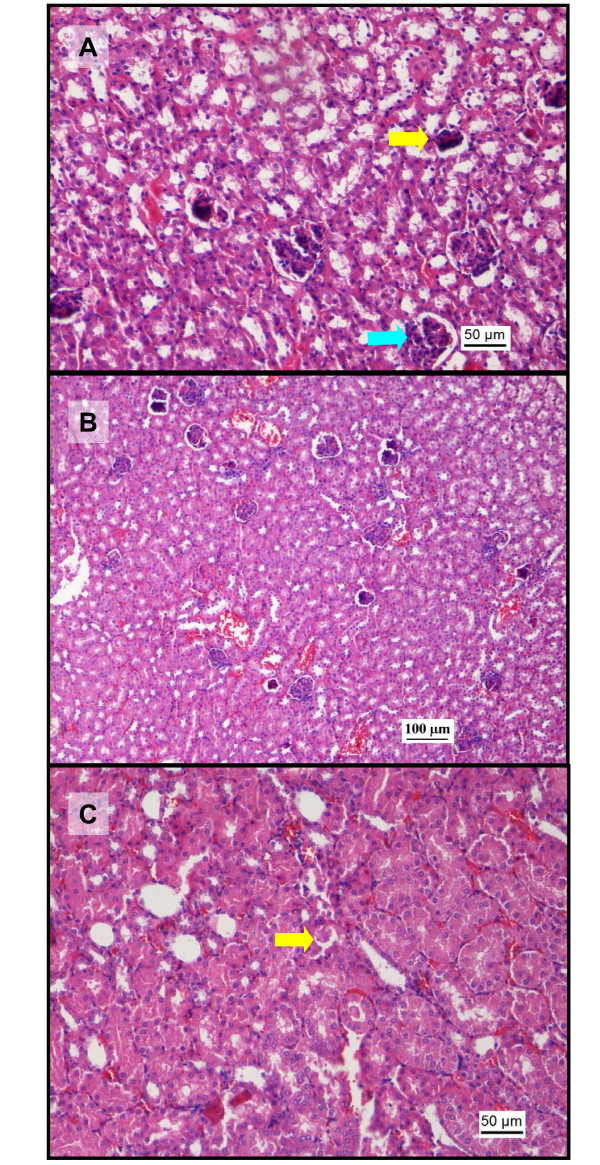
**Representative histopathological changes in kidney after administration of sunitinib alone or coadministered with paracetamol (H&E staining)**. Photomicrographs of kidney tissue sections after administration of sunitinib or sunitinib and paracetamol. A: (200x) photomicrograph after administration of sunitinib (50 mg/kg) showing glomeruli atrophy (yellow arrow). Notice the reduction in size and cellularity, when compared to normal glomeruli (blue arrow). B: photomicrograph (100x) after administration of sunitinib (80 mg/kg) showing glomerular atrophy admixed with congested spaces and extravasated red blood cells in the interstitium. C: photomicrograph (200x) after coadministration of sunitinib (140 mg/kg) and paracetamol, showing tubules filled with casts in the lumen (arrow).

**Figure 8:**
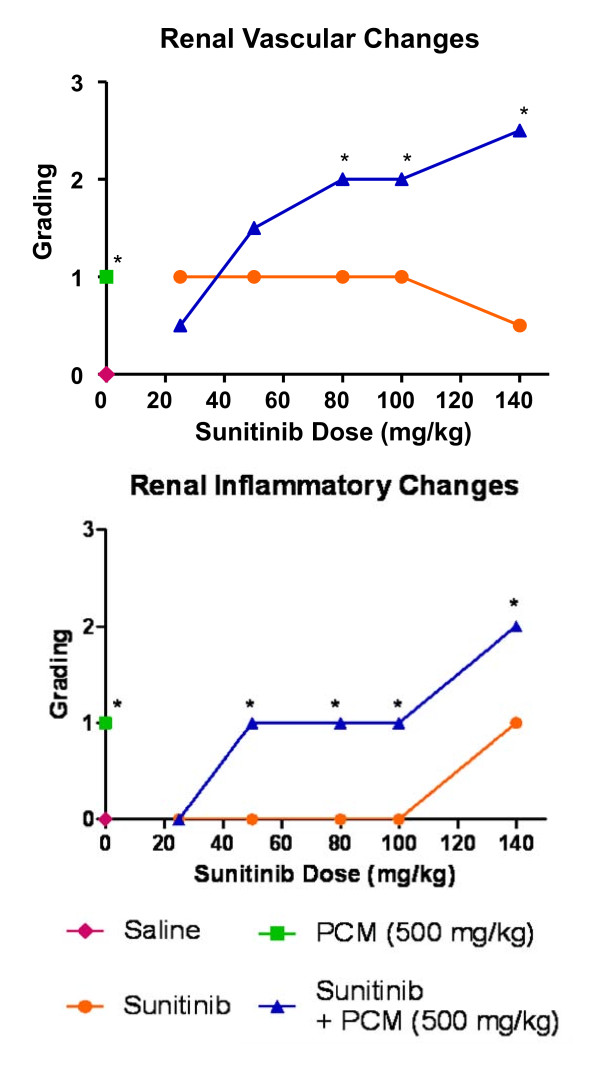
**Renal vascular and inflammatory changes**. Graphical representation of the score and grading of the vascular and inflammatory changes observed in renal tissue after administration of saline vehicle (baseline control), paracetamol, sunitinib at several doses or combination treatment paracetamol and sunitinib (* p < 0.05 bewteen groups C and D based on Mann Whitney test).

Normal histology of the glomerulus and tubules was found in kidney tissue of mice that received saline vehicle only (Figure 6). Paracetamol induced mild vascular and inflammatory changes with signs of vascular congestion, tubular necrosis and glomerular atrophy, which is a degenerative phenomenon (Figure 6). Mice treated with sunitinib alone only showed mild vascular changes in the kidneys comparable to those observed upon administration of paracetamol, but no signs of inflammatory changes were observed, except at the highest dose of sunitinib (140 mg/kg) where some tubular necrosis was noted (Figure 7). In the combination treatment group, the vascular changes increased (p < 0.05 above 80 mg/kg dose in comparison to group C) from mild to moderate damage as the sunitinib dose increased (Figure 7 and 8). However, the inflammatory changes were mild, similar to those in group B at low sunitinib doses and become moderate changes at the highest sunitinib dose (140 mg/kg) where tubular casts were observed (Figure 8) and were statistically significant above 50 mg/kg doses).

### Histopathology findings in heart, brain and spleen

**Figure 9:**
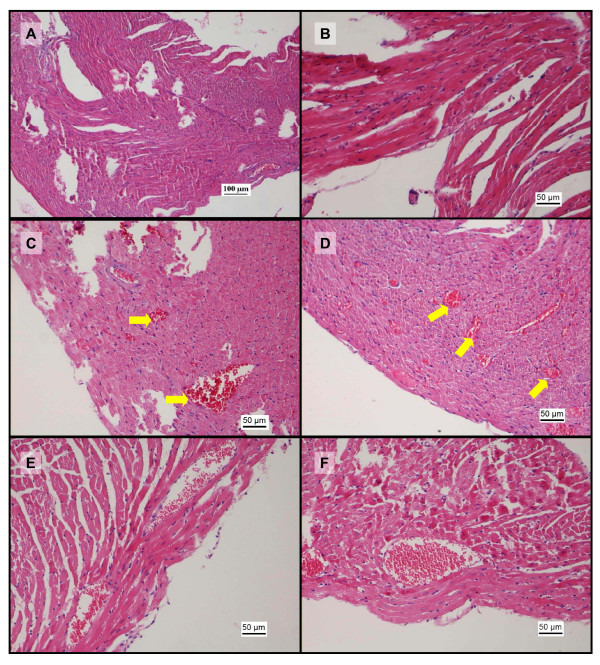
**Photomicrographs of representative histopathological changes in heart tissue (H&E staining)**. Photomicrographs of kidney tissue sections after administration of sunitinib or sunitinib and paracetamol. A: photomicrograph (100x) from heart tissue from the control saline group showing the ventricular wall with normal orientation of healthy cardiomyocytes. B: photomicrograph (200x) from the saline vehicle group showing the normal morphology of the muscle fibers with abundant wavy cytoplasm and small nuclei. C: photomicrograph (200x) after administration of paracetamol showing numerous congested vessels (arrows) amidst the muscle fibres. D: photomicrograph (200x) showing congested and dilated blood vessels (arrows) in the heart muscle filled with red blood cells after administration of 50 mg/kg sunitinib. E: photomicrograph (200x) showing congested and dilated blood vessels in the heart muscle after coadministration of 80 mg/kg sunitinib with paracetamol. F: photomicrograph (200x) showing further congested and dilated blood vessels in the heart muscle and filled with red blood cells after coadministration of sunitinib (100 mg/kg) and paracetamol.

**Figure 10:**
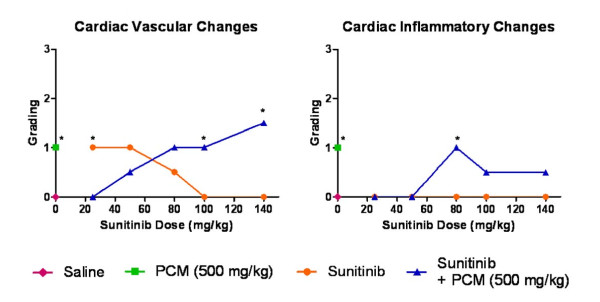
**Vascular and inflammatory changes in heart tissue**. Graphical representation of the score and grading of the vascular and inflammatory changes observed in cardiac tissue after administration of saline vehicle (baseline control), paracetamol, sunitinib at several doses or combination treatment paracetamol and sunitinib at several doses (* p < 0.05 bewteen groups C and D based on Mann Whitney test).

The heart tissues from the vehicle control group showed normal cardiomyocytes with no vascular or inflammatory changes (Figure 9). The cardiovascular tissue from mice treated with paracetamol showed mild vascular congestion and inflammatory changes such as myocyte coagulation (Figure 9). Sunitinib treatment resulted in mild vascular congestion (Figure 9) but no inflammatory changes were noted. The histopathology of the heart in the coadministration treated mice showed moderate vascular and inflammatory changes at 100 and 140 mg/kg which were significant (p < 0.05) and included congested and dilated blood vessels (Figure 9). However, mice treated with the lower doses of sunitinib alone showed reduction in cardiotoxicity in relation to the paracetamol group, which ranged from no damage to moderate damage at the 25 - 80 mg/kg doses of sunitinib (Figure 10).

**Figure 11:**
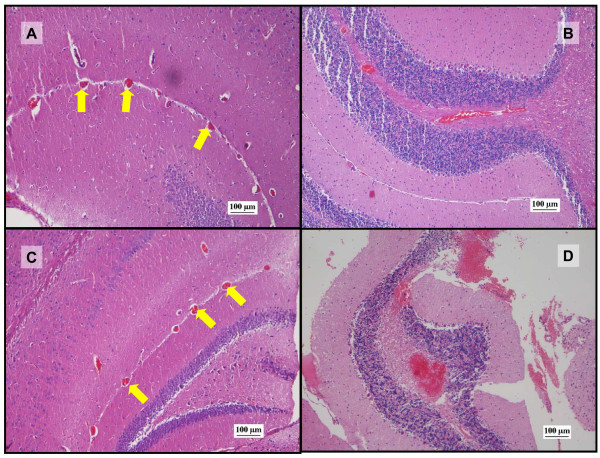
**Photomicrographs of representative histopathological changes in brain tissue (H&E staining, 100x)**. Photomicrographs of representative brain regions after administration of sunitinib or combination treatment of sunitinib and paracetamol. A: photomicrograph showing zones of congested blood vessels of moderate to small size (arrows) bordering the cellular areas after administration of sunitinib 50 mg/kg dose. B: photomicrograph from the sunitinib 80 mg/kg group showing vascular congestion in the brain. C: photomicrograph from the combination treatment sunitinib (50 mg/kg) and paracetamol group showing vascular congestion (arrows) in the brain. D: photomicrograph showing vascular congestion in the brain after coadministration of sunitinib 100 mg/kg and paracetamol.

**Figure 12:**
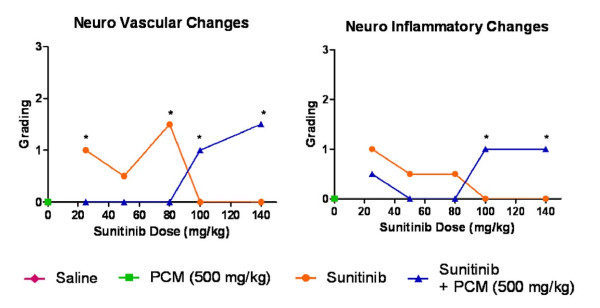
**Vascular and inflammatory changes in brain**. Graphical representation of the score and grading of the vascular and inflammatory changes observed in brain tissue after administration of saline vehicle (baseline control), paracetamol, sunitinib at several doses or combination treatment paracetamol and sunitinib at several doses (* p < 0.05 bewteen groups C and D based on Mann Whitney test).

The neurohistopathology assessment demonstrated normal neuronal cells with no vascular or inflammatory changes after administration of saline or paracetamol. Some congestion of vessels at the 'cortical junction' as well as evidence of liquefactive necrosis was observed in mice treated with sunitinib alone at low doses (p < 0.05 at 25 and 80 mg/kg) but the toxicity decreased at high doses (Figure 11). The combination treatment group D, overall, showed less neurotoxicity than group C at low doses of sunitinib with lesser vascular congestion but a raise of neurotoxicity including generalised congestion was seen at high doses (p < 0.05), of sunitinib in combination with paracetamol (Figure 12).

**Figure 13:**
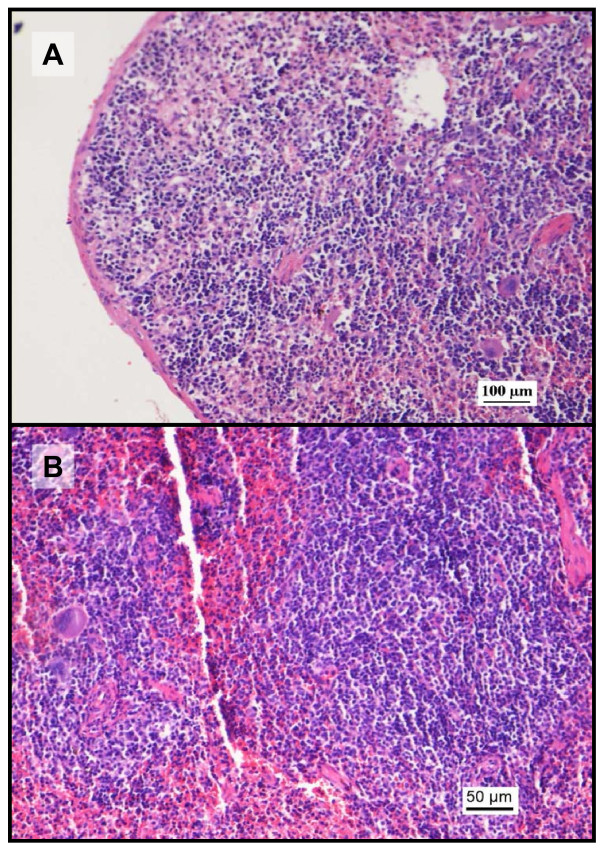
**Photomicrographs of the histopathology of the spleen (H&E staining)**. Histopathological changes were observed only at the highest dose of sunitinib (140 mg/kg) coadministered with paracetamol. A: photomicrograph (100x) from the control saline group showing normal morphology of the spleen including the red and white pulp areas. B: photomicrograph (200x) after coadministration of sunitinib 140 mg/kg and paracetamol showing areas of cellularity admixed with mild congestion.

Finally, the histology assessment in the spleen did not reveal any vascular changes in the paracetamol treated group, sunitinib treated mice or the sunitinib-paracetamol drug combination group. Only a very slight splenic congestion was observed at the highest dose of 140 mg/kg (p < 0.05) sunitinib-paracetamol combination (Figure 13). Furthermore, no inflammatory changes were observed at any dose in the sunitinib alone group, paracetamol alone group, or in the sunitinib plus paracetamol drug combination group.

## Discussion

The histopathological changes in the liver, kidneys, heart, brain and spleen together with hepatic and renal function biochemical markers were evaluated after coadministration of sunitinib with paracetamol. Clinical trials in mRCC patients had shown that liver and renal functions are affected by sunitinib leading to elevated AST, ALT and creatinine plasma levels [11]. Furthermore, the wide-spread use of paracetamol in cancer patients as an effective pain management drug with its potential renal and hepatotoxicity may elevate the risk of organ dysfunction after coadministration [10], a phenomenon observed with other anticancer drugs [9].

### Possible hepatoprotective effect of sunitinib

In the current study, a significant rise in serum AST and ALT levels was observed after administration IP of 500 mg/kg paracetamol dose in comparison with the baseline control group (Figure 1). This is a consistent finding with previous studies where it was shown that the rise in serum AST and ALT concentrations peak 4 h after administration of 500 mg/kg paracetamol IP [36]. In addition, the histopathological analysis also showed vascular and inflammatory changes (Figure 2) such as centrilobular necrosis, congested sinusoids and nuclear changes which were similar to other studies [35]. In contrast, after sunitinib was administered at escalating doses, the serum AST and ALT levels were only slightly elevated and mild liver damage with slight vascular congestion and signs of hepatocyte regeneration were found (Figure 3). No studies have yet been conducted on the histopathology changes caused by sunitinib administration; however, it was observed in clinical studies conducted on patients receiving sunitinib for the treatment of GIST or mRCC a slight increase in ALT and AST serum concentrations [11,12]. In addition, the combination treatment group showed. lower AST and ALT serum levels in comparison to group B (paracetamol, 500 mg/kg, IP) at low doses of sunitinib coadministered with paracetamol. However, at higher doses of sunitinib in group D, both the AST and ALT levels were significantly increased (Figure 1). The histopathological analysis of the hepatic tissue showed a similar trend. At low doses of sunitinib coadministered with paracetamol, the hepatic morphological changes showed less severe damage compared to group B. Meanwhile, at higher doses of sunitinib (group D), a greater severity of the morphological changes such as vascular congestion, fatty change, and centrilobular necrosis were observed, indicating increased toxicity (Figure 5).

The biochemical and histopathological findings of the liver were unexpected. Low doses of sunitinib and paracetamol in the coadministration group seemed to decreased paracetamol hepatotoxicity, suggesting that sunitinib may have some level of hepatoprotective effect as previously suggested [10]. However, in the present study, the protective effect seemed to be obliterated at higher doses of sunitinib coadministered with paracetamol.

Therefore, a preliminary mechanistic working-hypothesis based on the paracetamol metabolic pathway may be postulated in the attempt to explain the differential effect between the low and the high doses of sunitinib upon coadministration with paracetamol. The metabolism of paracetamol is mediated by CYP2E1, 1A2 and 3A4 to form the toxic metabolite NAPQI [37], while sunitinib is metabolized by CYP3A4, 1A1 and 1A2 to form the active metabolite SU12662 [13,30]. Thus, as both sunitinib and paracetamol share the same isoenzyme CYP3A4 for their metabolism, the generation of NAPQI from this pathway may be decreased, resulting in less toxic effects. However, as the dose of sunitinib coadministered with paracetamol is increased, the protective effect is reversed and greater toxicity is observed. This could be explained if sunitinib or its metabolite affect the glutathione mediated detoxification pathway of paracetamol, either by binding to glutathione or by decreasing its available pool reducing the capacity to protect the hepatocytes from the electrophilic damage caused by NAPQI [38]. Then, even after lower generation of NAPQI , the depletion of glutathione available would lead to NAPQI accumulation and toxicity. Although conjugation of sunitinib with glutathione is not reported in the literature, sunitinib is a substrate and a competitive inhibitor of a glutathione-conjugate transporter and may decrease the availability of glutathione in the hepatocytes [39]. A similar mechanistic explanation has been proposed for cisplatin, a chemotherapy drug which binds to glutathione [40]. Although the current experiment was not designed to evaluate the potential hepatoprotective effect of sunitinib in a paracetamol induced liver toxicity model, the results point out towards a certain protective effect as observed previously (10). Further studies need to be conducted to clarify the intertwining relationship between paracetamol, sunitinib, the formation of NAQPI and the glutathione cellular concentration.

### Renal toxicity

The biochemical markers BUN and creatinine were used to evaluate renal function. Administration of 500 mg/kg paracetamol IP caused a significant rise in BUN concentrations (Figure 1) which was consistent with other studies after oral [41] or IP administration [42] of paracetamol, although the biomarker concentrations were measured at a later time point (24 h). The toxicity associated with paracetamol also resulted in glomerular atrophy and necrosis of the tubules, similar to those observed in acute tubular necrosis in both proximal and distal parts of the tubules including damage to the glomerulus (Figure 6) [41,43]. Administration of sunitinib caused a small elevation of BUN levels and mild vascular congestion at the highest dose (140 mg/kg). When sunitinib was coadministered with paracetamol, the BUN plasma levels and the histopathological analysis at low doses of sunitinib were similar to those of paracetamol treated group suggesting that the toxicity may be due to the presence of paracetamol only. Besides, the circulating paracetamol toxic metabolite may be generated in the liver as well at the proximal tubule of the kidney by the CYP2E1 enzyme [44]. However, at higher doses of sunitinib, the BUN plasma concentrations rose above those of observed in the groups that were given paracetamol or sunitinib alone and was accompanied by increased vascular and inflammatory changes (Figure 8). This suggests that the renal toxicity observed at higher doses may be a combined effect of the toxicity contributed by sunitinib and paracetamol.

A significant correlation between the BUN levels and kidney morphological changes was present (Table 2). However, the creatinine biomarker did not show any correlation with the histopathological findings. This finding is similar to other studies where no significant rise in plasma creatinine level was noted 6 h after paracetamol administration at higher doses [45]. Thus, creatinine plasma levels may not be an early indicator of renal toxicity, even though histopathological changes were observed in the kidneys [46,47].

**Table 2:** Significant values of the correlation between biomarkers and liver and renal histopathological changes based on Spearman rank correlation test.

Biomarker	Liver changes		Kidney changes	
	
	Vascular	Inflammatory	Vascular	Inflammatory
AST	p < 0.01	p < 0.01	n/a	n/a
ALT	p < 0.01	p < 0.01	n/a	n/a
BUN	n/a	n/a	p < 0.01	p < 0.01
Creatinine	n/a	n/a	p > 0.05	p > 0.05

The correlation is significant if p < 0.01.

The protective effect of sunitinib on paracetamol toxicity was not as obvious in the kidneys as it was in the liver. This may be due to additional mechanisms contributing to paracetamol toxicity such as prostaglandin synthetase and N-deacetylase enzymes [29], different mechanisms and sensitivity of the renal tissue [48] or different *in situ *biotransformation [49]. Consequently, sunitinib may not be able to affect the paracetamol metabolic pathway in the kidneys.

### Associated toxicity in heart, spleen and brain

Several studies carried out in humans with sunitinib have reported signs of toxicity in other organs besides the liver or the kidneys [50].

In the present study, sunitinib treatment showed vascular congestion in the heart with no inflammatory changes in the cardiomyocytes (Figure 9). These results are consistent with observations in clinical studies where the cardiotoxicity did not present inflammatory or fibrotic changes in patients [23]. Some of the mechanisms of cardiotoxicity for other tyrosine kinase inhibitors have been determined, but the mechanism of cardiotoxicity due to sunitinib is yet to be discovered [51]. Although paracetamol cardiotoxicity is rare, non-specific ECG changes, bradycardia, pericardial rub and endocarditis, were observed in patients [52]. Furthermore, subendocardial haemorrhages and muscle necrosis was noted in the autopsies of two patients who died from paracetamol overdose [53]. Mice in the paracetamol treatment group showed signs of toxicity with congested vessels and focal myocyte coagulation (Figure 9). It has been suggested that these toxicity pattern may be associated with paracetamol metabolite and free radical NAPQI, resulting in myocyte glutathione depletion, damage to the myocardium and breakdown of endothelium-derived vascular relaxing factor (EDRF), leading to functional coronary insufficiency [33] and myocyte coagulative necrosis which has been observed in our study and in previous ones [52]. The combination treatment also showed dose dependent cardiac toxicity with a pattern similar to that observed in liver: At low doses of sunitinib in the drug combination, the scoring of the morphological changes was below that of paracetamol alone, while the toxicity increased slightly at the higher doses of sunitinib with paracetamol. Extrahepatic paracetamol toxicity in kidneys, lungs and nasal glands has been attributed to circulating NAPQI generated in the liver [49]. Thus, it is possible to suggest that the cardiotoxicity may be associated with the generation of toxic metabolite by the liver and would follow the liver pattern of toxicity as proposed with the working hypothesis [36]. Further studies, including cardiac biomarkers such as CK-MB and quantification of circulating NAPQI are needed to assess sunitinib effects on the cardiac toxicity.

There was no evidence of neurotoxicity associated with paracetamol [54]. However, sunitinib caused vascular and inflammatory changes including vascular congestion and early signs of liquefactive necrosis (Figure 11). Previous animal studies have confirmed that sunitinib and its metabolite penetrate the brain up to 30%-40% of plasma concentrations in monkeys [55], 4-7 times the plasma concentration in mice [21]. In fact, some patients developed cognitive and behavioural changes during sunitinib treatment that included disorientation, confusion and word-finding difficulties [56] and posterior reversible encephalopathy syndrome in one patient [27]. These symptoms were reversible upon sunitinib discontinuation [27,56] but the mechanisms leading the development of neurological symptoms due to tyrosine kinase inhibition remain unknown [54]. At low doses of sunitinib coadministered with paracetamol, the morphological changes were lesser to those observed in the sunitinib alone treatment group which is consistent with previous findings suggesting anti-oxidant and anti-inflammatory effect of paracetamol on the cerebrovasculature due to menadione-induced oxidative stress [57]. Furthermore, the decreased toxicity observed in this study may be supported by lower sunitinib brain concentration after coadministration with paracetamol observed in a separate study [58,59]. However, when high doses of sunitinib were administered with paracetamol, the vascular congestion severity increased and liquefactive necrosis was noted suggesting other mechanisms of toxicity which are unknown.

Finally, there were no signs of toxicity in the spleen in all groups except at 140 mg/kg of sunitinib coadministered with paracetamol which mild splenic congestion was observed (Figure 13). Similar splenic congestion was also observed after barbiturate administration in dogs [60]. The absent of toxicity, except at a very high dose, may encourage the development of advanced particulate delivery systems to target specific sunitinib-sensitive tumours without the risk of toxicity associated to their accumulation in the spleen [61,62].

### Correlation between biomarkers and histopathological findings

Of especial interest is the ability to establish a correlation between toxicology and biomarkers that can provide early detection of toxicity and allow prompt intervention. In this study, we found a significant correlation (p < 0.01) between the liver biochemical markers (ALT and AST) and the liver histopathology for both vascular (p < 0.01) and inflammatory (p < 0.01) changes (Table 2). A significant correlation was also found between the BUN plasma levels and kidney morphological changes be it vascular (p < 0.01) or inflammatory (p < 0.01) changes. This suggests that ALT and AST may be used as early indicators for the detection of acute liver damage as well as BUN for renal toxicity in the treatment of sunitinib and paracetamol.

However, there was no correlation with creatinine plasma levels. This was found consistent with other studies where no significant rise in plasma creatinine level was noted 6 hours after paracetamol administration at higher dose [44]. Thus, this finding suggests that unlike BUN, creatinine may not be an early indicator of renal toxicity or that 4 hours is too early to observe any significant rise in creatinine levels [30,45].

## Conclusions

The present study has found changes in the toxicity pattern with the coadministration of paracetamol with sunitinib compared to sunitinib alone.

Sunitinib administration resulted in mild toxicity in the liver, kidneys, heart and brain, asserting its safety at low doses. The coadministration of sunitinib and paracetamol had dose and differential effects in liver, kidneys and heart. However, no toxicity was observed in the spleen and neurotoxicity was observed only when sunitinib was administered alone. Sunitinib also seemed to display dose dependent, protective effects on paracetamol toxicity, especially regarding hepatotoxicity, which may be related to the fact that both paracetamol and sunitinib share the biotransformation pathway.

A significant correlation between the liver biomarkers (ALT and AST) and hepatic histopathological changes was found as well as between the renal biomarker BUN and kidney morphological changes. However, creatinine levels did not show a correlation with any study group. This suggests that ALT, AST and BUN may be valid early indicators for monitoring emerging toxicity in patients undergoing treatment with sunitinib and paracetamol.

## Competing interests

The authors declare that they have no competing interests.

## Authors' contributions

All authors contributed to the development of the project, the realization, the critical analysis and interpretation of the results. All authors reviewed and approved the manuscript.
